# Intramedullary screw fixation for simple displaced olecranon fractures

**DOI:** 10.1007/s00068-019-01114-4

**Published:** 2019-03-16

**Authors:** Willem-Maarten P. F. Bosman, Benjamin L. Emmink, Abhiram R. Bhashyam, R. Marijn Houwert, Jort Keizer

**Affiliations:** 1 Department of Surgery, St Antonius Hospital, Utrecht, The Netherlands; 2 Department of Surgery, Albert Schweitzer Hospital, Dordrecht, The Netherlands; 3 Department of Trauma Surgery, University Medical Center, Utrecht, The Netherlands; 4 Harvard Combined Orthopaedic Residency Program, Harvard University, Boston, MA USA; 5 Harvard Medical School, Boston, MA USA

**Keywords:** Olecranon fracture, Intramedullary screw

## Abstract

**Purpose:**

Olecranon fractures are common and typically require surgical fixation due to displacement generated by the pull of the triceps muscle. The most common techniques for repairing olecranon fractures are tension-band wiring or plate fixation, but these methods are associated with high rates of implant-related soft-tissue irritation. Another treatment option is fixation with an intramedullary screw, but less is known about surgical results using this strategy. Thus, the purpose of this study was to report the clinical and functional outcomes of olecranon fractures treated with an intramedullary cannulated screw.

**Methods:**

We identified 15 patients (average age at index procedure 44 years, range 16–83) with a Mayo type I or IIA olecranon fracture who were treated with an intramedullary cannulated screw at a single level 2 trauma center between 2012 and 2017. The medical record was reviewed to assess radiographic union, postoperative range of motion and complications (including hardware removal). Patient-reported outcome was evaluated using the Disabilities of the Arm, Shoulder and Hand (DASH) score. Average follow-up was 22 months (range 8–36 months).

**Results:**

By the 6th month post-operative visit, 14 patients had complete union of their fracture and 1 patient had an asymptomatic non-union that did not require further intervention. Average flexion was 145° (range 135–160) and the average extension lag was 11° (range 0–30). Implants were removed in 5 patients due to soft-tissue irritation. Average DASH score (± standard deviation) by final follow-up was 16 ± 10.

**Conclusions:**

Fixation of simple olecranon fractures with an intramedullary screw is a safe and easy fixation method in young patients, leading to good functional and radiological results. Compared to available data, less hardware removal is necessary than with tension-band wiring or plate fixation.

## Introduction

Olecranon fractures are relatively common injuries and account for approximately 10% of upper extremity fractures in adults [1]. Traction of the triceps on the proximal fragment often leads to disruption of articular congruity and of the elbow’s extension mechanism. As a result, this injury is typically treated with open reduction and internal fixation (ORIF) [1–5]. Common techniques to treat simple olecranon fractures are tension-band wiring and plate fixation [1–11]. As the skin is thin at the proximal ulna with relatively little subcutaneous tissue, these fixation methods often lead to implant-related soft-tissue irritation necessitating implant removal in 68–82% of the cases largely based on the fixation method that was used [3–5, 12].

**Fig. 1:**
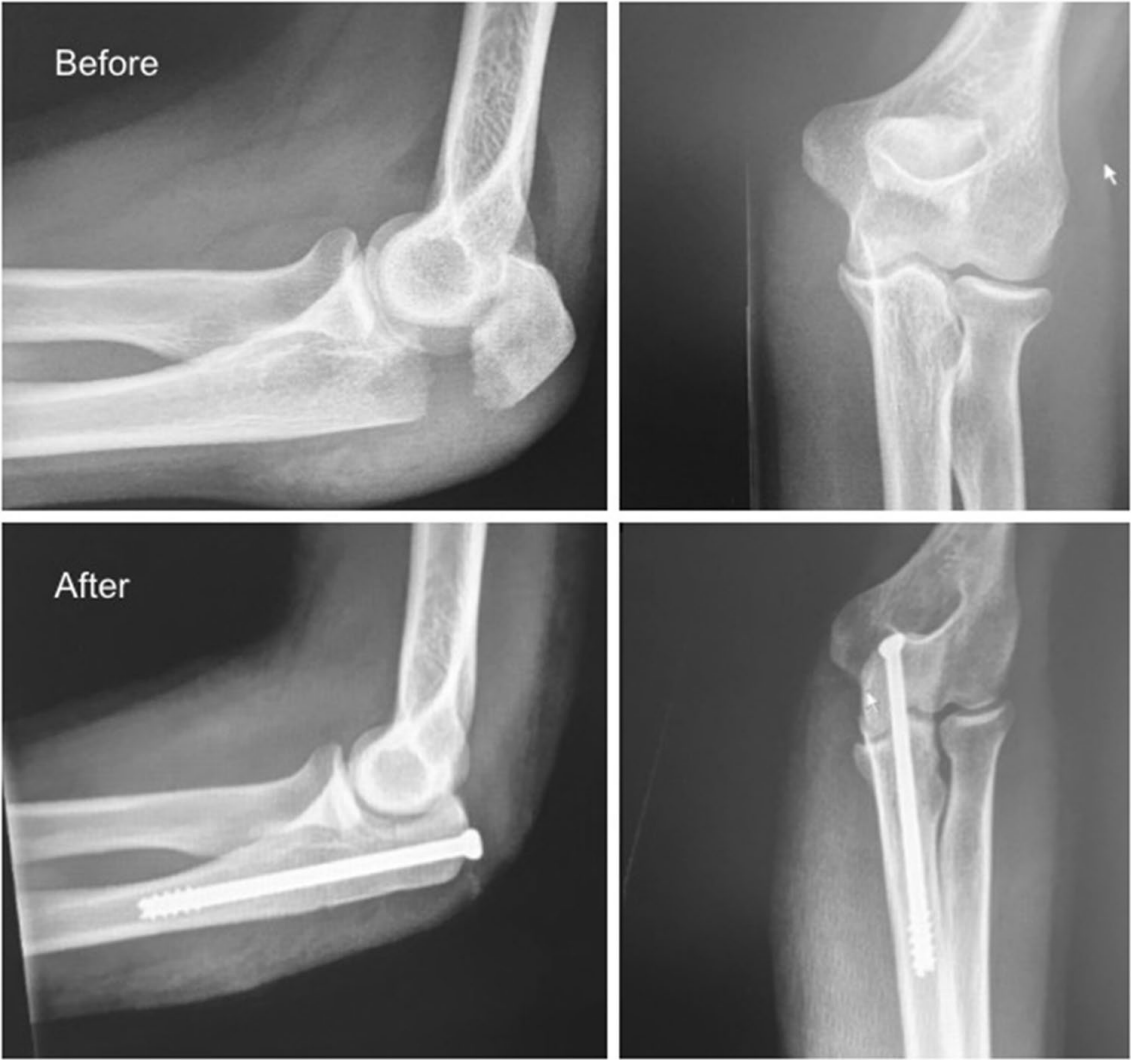
Displaced simple olecranon fracture, before and after fixation with a cannulated intramedullary screw

While intramedullary screw fixation is commonly used to fix olecranon osteotomies [13, 14] fewer reports have been published on its use for simple (Mayo type I or IIA) olecranon fractures (Fig. 1). Successful fixation with an intramedullary screw was first described in 1942 by MacAusland, but subsequent reports noted that the technique was challenging and unreliable [11, 15–18].

However, many of these studies are older without clear inclusion criteria or CT imaging. As we have learned more about the morphology of the ulna and proximal ulna fractures [19–21], it is less clear if early reports about the unreliability of intramedullary screw fixation were related solely to implant choice or to the use of an intramedullary screw for inappropriate (i.e., more complex) fracture types [22]. Thus, the purpose of this study was to assess the clinical and functional outcomes of simple olecranon fractures treated with an intramedullary cannulated screw.

## Methods

### Study design and inclusion criteria

For this retrospective study, we used billing records of a single level 2 trauma center to identify patients with a proximal ulna fracture treated between June 2014 and June 2017. The medical records and radiology images of these patients were reviewed: extra-articular ulna fractures, combined forearm fractures, comminuted olecranon fractures and all patients treated conservatively or with tension-band wiring or plate fixation were excluded. Based on these restrictions, we identified 15 patients (age > 16 years) with a Mayo type I or IIA olecranon fracture that was treated with an intramedullary 7.3 mm cannulated screw.

### Surgical technique

**Fig. 2:**
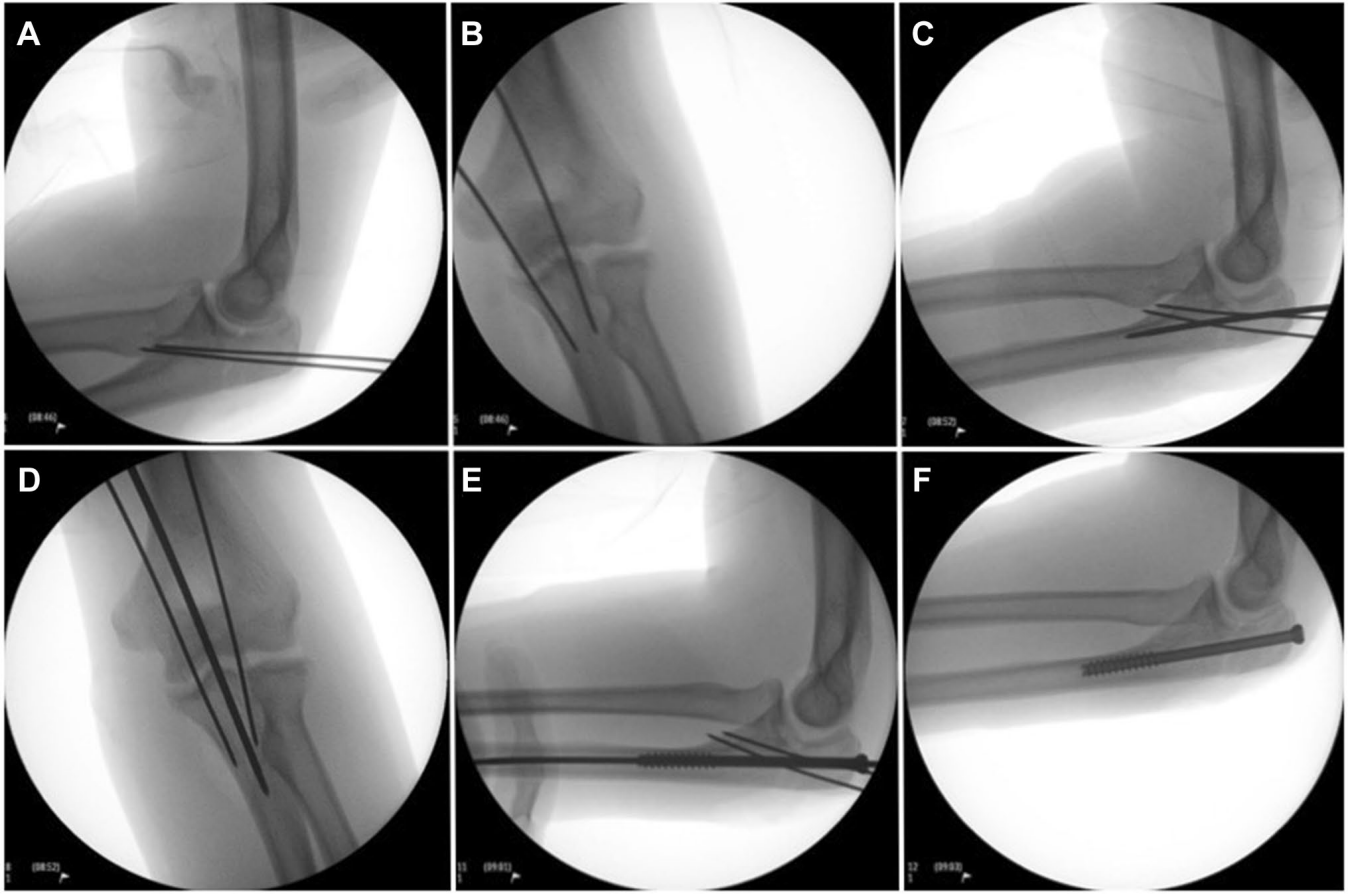
Overview of intra-operative fluoroscopy imaging: temporary reduction with bicortical anti-rotation k-wires (**a, b**). intramedullary placement of guide wire centre–centre on the olecranon tip into the ulnar shaft (**c, d**). Placement of the partially threaded cannulated screw over the guide wire (**e**). Final imaging after removal of the guidewire and the anti-rotation k-wires (**f**)

The patient is placed in the supine position with the arm draped over the chest. A straight posterior incision is made from the tip of the olecranon to 1 cm distal of the fracture. The fracture is irrigated and then reduced anatomically. Provisional fixation is obtained with two parallel bicortical 1.0 mm k-wires that are placed in such a way that they will not hinder the placement of the 7.3 cannulated intramedullary screw (Fig. 2a, b). We use two k-wires for provisional fixation to prevent rotation of the proximal ulna during placement of the screw. A small longitudinal incision is made at the triceps insertion over the centre–centre point of the palpable olecranon tip to facilitate placement of an intramedullary 2.8 mm guide wire (Fig. 2c, d) [23]. Screw length is then measured such that the distal threaded end of the screw will engage the narrow marrow of the proximal ulnar diaphysis (typically 90–110 mm) to provide stable fixation. The cortex is opened with the cannulated drill and the screw is placed. Depending on surgeon preference, an additional washer can be used (Fig. 2e). The guidewire and the two anti-rotation k-wires are then removed (Fig. 2f).

### Postoperative management and follow-up

The arm is placed in a padded bulky dressing for the first 24 h. All patients are allowed full active and passive range of motion, but are limited to non-weightbearing activities for the first 6 weeks. Patients are then seen at the outpatient clinic at regular intervals (2, 6, 12 and 24 weeks) until radiographic healing is observed.

### Evaluation

We recorded radiographic union, postoperative range of motion and complications (including hardware removal). Radiographic union was based on the most recently available radiograph. Active range of motion and implant-related complaints were noted in the electronic medical record. Patient-reported outcome was evaluated using the Disabilities of the Arm, Shoulder and Hand (DASH) score (including the Sport and Work sections) [24]. In general, the DASH ranges from 0 (no disability) to 100 (most severe disability), with lower scores denoting better function. In this study, we used the Dutch version of the DASH. We also assessed operating time as a proxy for procedure complexity.

### Statistical analysis

Data were collected in a Microsoft Excel 2016 database. Descriptive statistics were calculated for demographic characteristics, range of motion, complications, and patient-reported outcome (DASH). This study was approved by our Institutional Review Board.

## Results

### Study population

We identified 15 patients with a simple olecranon fracture (Mayo IA/IIA) that was treated with a 7.3 mm cannulated intramedullary screw. Fourteen of the 15 fractures were displaced (Mayo IIA) and 1 fracture was slightly displaced (Mayo IA). Average age of these patients was 44 years (range 16–83) years (Tables 1, 2). Ten patients were ASA I, 4 were ASAII and 1 patient was ASA III at their pre-operative screening. A washer was used in 10 patients, while 5 patients were treated with just a screw. Average duration operative time was 46 min (median = 41 min; range 35–72 min).

**Table 1:** Patient demographics

Patients	
N	15
Age (± SD)	44 ± 19
ASA I	10
ASA II	4
ASA III	1
Fracture	
Mayo 1a	1
Mayo 2a	14
Treatment	
Cannulated screw	5
Cannulated screw with washer	10
Operating time in minutes (± SD)	46 ± 9

**Table 2:** Overview of patients including age, fracture type, treatment modality, ASA classification, bony healing, function, and hardware removal

Age	Mayo	Treatment	ASA	Union	ROM (F/E)	Hardware removal	DASH
75	2A	Screw	2	Malunion	150-10-0	No	16
16	1A	Screw	1	Union	150-0-0	No	0
19	2A	Screw	1	Union	150-10-0	No	14
83	2A	Screw and washer	3	Nonunion	120-30-0	No	25
56	2A	Screw and washer	2	Union	150-10-0	No	39
51	2A	Screw	1	Union	150-0-0	No	19
23	2A	screw	1	Union	150-0-0	Yes	NA
57	2A	Screw and washer	1	Union	135-10-0	No	25
36	2A	Screw and washer	2	Union	160-10-0	Yes	0
34	2A	Screw and washer	1	Union	110-20-0	Yes	7
58	2A	Screw and washer	1	Union	150-10-0	No	17
16	2A	Screw and washer	1	Union	150-0-0	Yes	NA
73	2A	Screw and washer	2	Union	135-20-0	No	30
24	2A	Screw and washer	1	Union	130-20-0	No	NA
46	2A	Screw and washer	1	Union	160-10-0	Yes	0

*NA* not applicable

### Surgical results

**Fig. 3:**
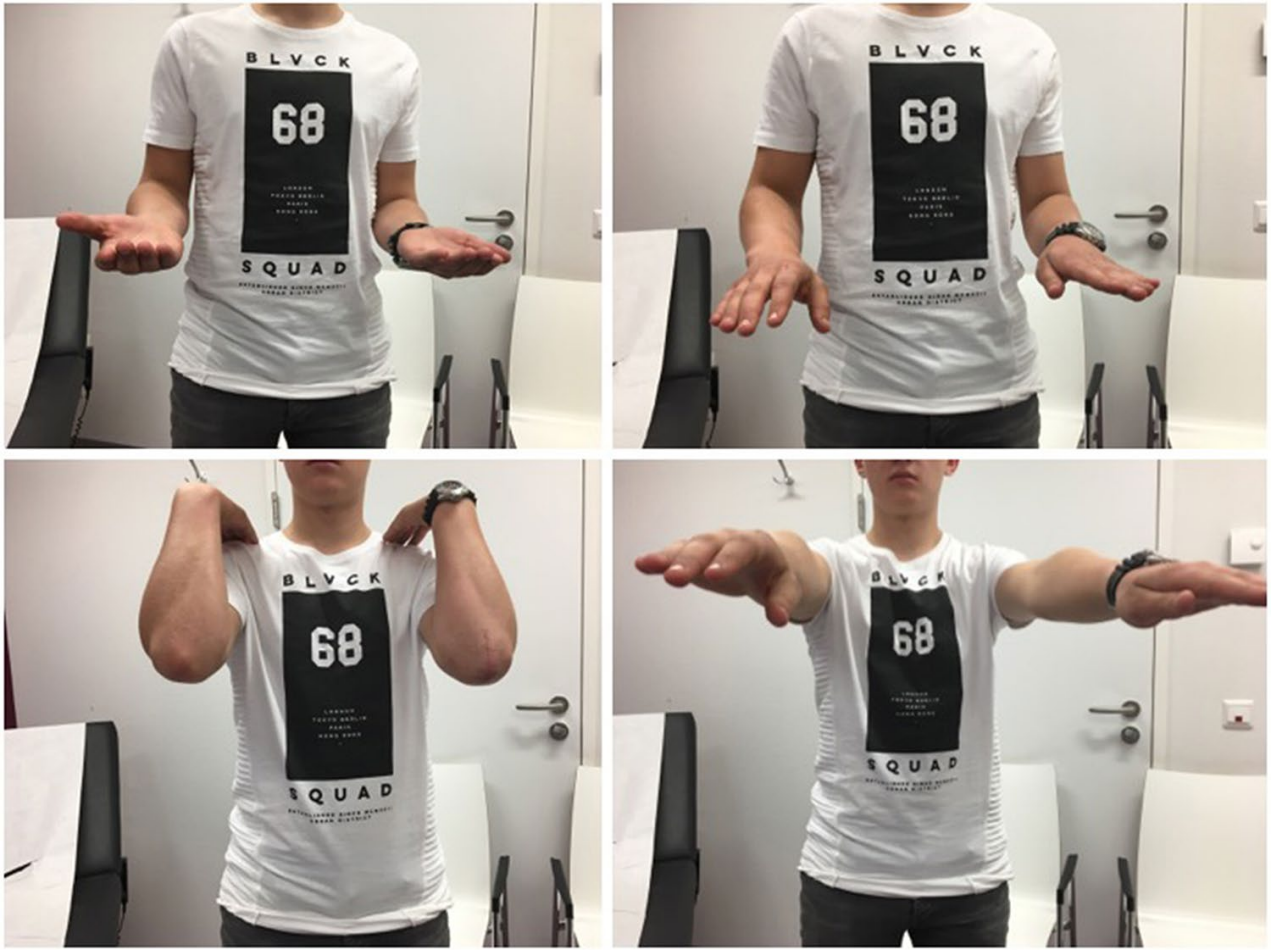
Function, 6 weeks after fixation with cannulated screw: full range of motion

The majority (14 out of 15) of fractures treated with intramedullary screw demonstrated healing by the time of final follow-up. One patient developed an asymptomatic non-union and another patient had an asymptomatic malunion; both were the oldest patients in the cohort and did not require any surgical intervention (Table 2). None of the patients required revision surgery. Average flexion was 145 ± 10°, average extension was 11 ± 6°, and average active range of motion was 134 ± 16° (Table 3; Fig. 3). Except for the patient with a non-union, all patients had at least 30° extension, 130° flexion, and at least a 100° flexion–extension arc (Table 3).

**Table 3:** Result at follow-up

Fracture healing: *n* (%)	
Bony healing	14 (93.3%)
Non-union	1 (6.7%)
Malunion	1 (6.7%)
Hardware-related complaints	5 (33.3%)
Re-operations: *n* (%)	
Revision surgery	0 (0%)
Hardware removal	5 (33.3%)
Function: (average ± SD)	
Flexion in degrees	145 ± 10°
Extension in degrees	11 ± 6°
Active range of motion	134 ± 16°
DASH scores: (average ± SD)	12 of the 15 returned
DASH DLV (12/15)	16 ± 10
DASH work module (9/15)	7 ± 10
DASH sports module (7/15)	27 ± 20

### Complications

Other than complications related to radiographic healing, the only complication observed in this series was removal of implants due to implant-associated soft-tissue irritation in 5 patients. Interestingly, 4 out of 10 patients treated with a washer required implant removal, while only 1 out of 5 patients treated with screw only required removal. The average time ± SD to hardware removal was 332 ± 206 days.

### Patient-reported outcome

DASH scores were available for 12 out of 15 patients (80% response rate). Average DASH scores were 16 ± 10. Average scores on the DASH work module were 7 ± 10, while average scores on the DASH sports module were 27 ± 20 (Table 2).

## Discussion

While tension-band wiring and ORIF with plate fixation are both effective treatments for olecranon fractures, we propose that fixation of simple olecranon fractures using an intramedullary screw is also a reasonable option based on the results of this series. Previous studies have suggested that fixation using intramedullary cannulated screws is technically challenging and unreliable [11, 15–18], but we found that this technique lead to acceptable surgical results, good patient-reported outcomes, and low rates of implant removal with reasonable operative times (average 45 min).

When surgical treatment is indicated, several treatment options, such as tension-band wiring, plate fixation, intramedullary screw fixation and intramedullary nail fixation have been described [1–5, 7–11]. These techniques differ in their method of fixation and have their own advantages and disadvantages, but a recent Cochrane review found that none was definitively preferable based on available evidence [2]. Tension-band wiring has shown excellent results with regard to union rates [2, 3, 9] and remains the first choice of treatment for many due to the low complexity and costs of the [8, 9]. Yet, implant removal is often necessary due to hardware irritation or k-wire migration in up to 82% of patients [3, 7]. Another technique often used is plate fixation [5, 8, 9, 25]. This technique is especially indicated in comminuted fractures and with osteoporotic bone, but can also be used for simple two-part fractures [5, 8]. In a recent prospective trial comparing plate fixation and tension-band wiring, there was no difference in union, DASH, function or costs, and plate fixation was associated with a decreased rate of implant removal [3]. However, this was offset by the increased number of infections and revisions in the plate fixation group [3]. Since neither of these options is optimal for all settings, we propose that intramedullary screw fixation should also be considered for simple olecranon fractures based on our results.

Many reasons exist for differences between our study and existing literature [11, 15–17]. The prior studies are small and often include a wide variety of fractures treated with intramedullary screws, and frequently in settings where plate fixation would have been more appropriate given our greater understanding of proximal ulna fracture morphology [19–21]. For example, in the series published by Helm et al., 79% of treated fractures were comminuted [17]. In the present day, with more accurate radiologic diagnostics including improved X-rays and CT scans, identifying appropriate cases (e.g., simple olecranon fractures) is easier and likely to lead to better outcomes [22]. Moreover, using intramedullary screw fixation for simple Mayo type 1 and 2A fractures is somewhat intuitive since these fracture types are most similar to olecranon osteotomies which are known to heal well with screw fixation. And in many cases, use of a screw instead of tension-band wiring also led to decreased rates of implant removal [18].

Except for one elderly frail patient with an asymptomatic non-union, all patients healed their fracture. Mechanically, the partially threaded intramedullary screw works as a lag-screw. When inserted across the fracture, the threads of the screw tip engage in the cancellous bone of the metaphyseal area, causing compression of the fracture fragments upon tightening. In osteoporotic bone the screw may not have enough purchase for a stable fixation and the proximal part may be pulled away by the force of the triceps muscle. In our series, both the malunion and the non-union patient were geriatric patients with osteoporotic bone (Table 2). Based on these results, this fixation method may not be suitable for this patient group although this requires further study.

A prior study demonstrated that the dominant factor driving re-operation for isolated olecranon fractures was the type of fixation. In that study, tension-band wiring had over twice the rate of implant removal as ORIF using plates (46.5% vs. 18.7%, respectively), and rates using intramedullary implants were not studied [12]. Based on our results, intramedullary screw fixation has rates of removal that are in-between these two fixation methods. Other, earlier studies [3–5] have shown higher removal rates for tension-band wiring and plate fixation up to 68–82%. In addition, we did not observe any superficial or deep infections and this may have been due to the fact that, compared to tension-band wiring or plate fixation, a smaller incision could be used with minimal soft-tissue dissection or trauma. However, further studies with larger number of patients comparing between fixation options are needed to better answer these questions.

Finally, during the last 20 years several intramedullary nails have been described [10, 26, 27]. The nails have the same advantage as the intramedullary screw as they are low profile and have almost no protruding hardware. However, intramedullary nails are more complex and often designed for use with more complex fractures. For simple Mayo IA or 2A fractures, these extra design features are likely not necessary and simple screw fixation is likely to be sufficient and probably less expensive. We did not observe complications related to the insertion of a straight rigid intramedullary screw into a bone with a complex curved morphology. We believe this is because the varus curvature of the proximal ulna begins at approximately 8.2 cm from the olecranon tip and the anterior angulation starts at approximately 8.6 cm, while the inserted screws were only between 9 and 10 cm [28]. These anatomic characteristics highlight the importance of the central–central insertion point in facilitating the optimal direction of the screw [23].

### Limitations of study

This study has several limitations. As this was not a prospective randomized control trial, patient selection may be biased by the preference of the treating surgeons. However, our goal was to illustrate that this well-described technique may be more reliable than previously suggested. We had 100% follow-up rate for clinical exam and a 80% response rate to the DASH survey which minimized response bias. Finally, we studied a relatively small number of patients making it difficult to make robust statistical comparisons. However, given that this is an uncommon technique which we used for very specific indications, these results may help support the use of intramedullary screws at other institutions to allow for larger studies.

## Conclusion

Fixation of simple olecranon fractures using an intramedullary screw is a reasonable option, leading to acceptable surgical results, good patient-reported outcomes, and low rates of implant removal. Future work is needed to compare outcomes against other fixation options and to study other possible indications for its use.
